# Prevalence of efflux pump and heavy metal tolerance encoding genes among *Salmonella enterica* serovar Infantis strains from diverse sources in Brazil

**DOI:** 10.1371/journal.pone.0277979

**Published:** 2022-11-22

**Authors:** Felipe Pinheiro Vilela, Dália dos Prazeres Rodrigues, Marc William Allard, Juliana Pfrimer Falcão

**Affiliations:** 1 Faculdade de Ciências Farmacêuticas de Ribeirão Preto–USP, Departamento de Análises Clínicas, Toxicológicas e Bromatológicas, Ribeirão Preto, SP, Brazil; 2 Fundação Oswaldo Cruz–FIOCRUZ, Pavilhão Rocha Lima, Rio de Janeiro, RJ, Brazil; 3 Division of Microbiology, Office of Regulatory Science, Center for Food Safety and Applied Nutrition, U.S. Food and Drug Administration, College Park, Maryland, United States of America; University of Patras, GREECE

## Abstract

*Salmonella enterica* subspecies *enterica* serovar Infantis (*S*. Infantis) is a non-typhoid, zoonotic and foodborne serovar with worldwide distribution, and often associated with increasing antimicrobial resistance. Efflux pumps are antimicrobial resistance mechanisms able to promote and increase resistance levels to multiple distinct drug classes. Heavy metal tolerance genes have been demonstrated to promote resistance against these compounds and act in the co-selection of antimicrobial resistant strains. Despite the relevance of *S*. Infantis in clinical and non-clinical fields, few studies worldwide have investigated the occurrence of such genes in strains from diverse sources. Therefore, the present study aimed at determining the prevalence of antimicrobial efflux pump and heavy metal tolerance genes and their genomic relatedness through core-genome multi-locus sequence typing (cgMLST) of 80 *S*. Infantis strains isolated from food, environmental, human and animal sources from 2013 to 2018 in Brazil. Twenty efflux pump encoding genes were detected, with 17 of these (*acrA*, *acrB*, *baeR*, *crp*, *emrB*, *emrR*, *hns*, *kdpE*, *kpnF*, *marA*, *marR*, *mdtK*, *msbA*, *rsmA*, *sdiA*, *soxR* and *soxS*) detected in all strains studied, *golS* in 98.75%, *mdfA* in 58.75% and *tet(A)* in 37.5%. Tolerance genes to arsenic (*arsR*) were detected in 100% of the strains, gold (*golS* and *golT*) in 98.75%, silver (*silABCDEFPRS*) in 36.25% and mercury (*merR* and *merT*) in 1.25%. cgMLST demonstrated a closer genetic relationship among strains harboring similar profiles of heavy metal and efflux pump encoding genes, despite their origin. In conclusion, the high prevalence of some efflux pump and heavy metal tolerance encoding genes alert us about the importance of strong surveillance measures to monitor resistance and the transmission of *S*. Infantis among diverse sources in Brazil.

## 1. Introduction

Non-typhoid *Salmonella enterica* (NTS) serovars are among the four leading bacterial pathogens associated with human foodborne diseases in the world [1]. In association with its zoonotic nature, increasing antimicrobial resistance rates have been constantly reported about these pathogens, leading the U.S. Centers for Disease Control and Prevention (CDC) and the World Health Organization (WHO) to classify drug-resistant NTS as a serious threat and high priority pathogens in the control and prevention of antimicrobial resistance [2, 3].

*Salmonella enterica* subspecies *enterica* serovar Infantis (*S*. Infantis) is a non-typhoid serovar of global distribution and a ubiquitous nature, associated with human infections and present in foods, food-producing animals, farm and industry environments [4–6]. Increasing resistance rates have been reported for this serovar to drugs-of-choice employed in the treatment of human salmonellosis, such as fluoroquinolones and third- and fourth-generation cephalosporins, and drugs employed mainly in the veterinary field for animal therapy or as illegal growth promoters, such as aminoglycosides, phenicols and tetracycline [4–6].

In Gram-negative bacteria, such as NTS, antimicrobial resistance can be achieved by several types of mechanisms encoded by chromosomal mutations or horizontal transfer via plasmid-borne genes, which are usually responsible in conferring resistance to unique drug classes instead of multiple drug classes [7]. However, antibiotic-specific resistance genes are not the only ones capable of promoting drug resistance. Several genes encode the formation of efflux pumps in the cell membrane of Gram-negative bacteria, which are structures known for their capacity to promote and increase antimicrobial resistance levels to one or multiple distinct classes of antimicrobial molecules through pumping of the latter from the interior of bacterial cells to their exterior environment [8, 9].

Non-antibiotic compounds have also been demonstrated to play an important role in bacterial resistance. Heavy metals (such as arsenic, copper, mercury, silver and zinc) commonly occurr in nature, and some also are considered as environmental contaminants as a result of human pollution [10, 11]. Derivative products of these metals also have been employed in the treatment and prevention of bacterial infections in clinical fields, such as antiseptics or included in medical devices, and in non-clinical areas, such as disinfectants, food preservatives and feed additives for food-producing animals [10, 11]. Several genes have been reported to act in the bacterial tolerance to several types of heavy metals and also play an important role in the co-selection of antibiotic resistance strains [12].

In recent years, advancements in whole-genome sequencing (WGS) have significantly contributed to the study of zoonotic bacteria such as in the epidemiological monitoring of outbreaks and the tracking of antimicrobial resistance burden through genomics methods [13, 14]. Brazil is currently one of the world’s largest global meat exporters [15]. Despite this, and the common reports of *S*. Infantis in food items, environmental sources, humans and food-producing animals, few studies have employed genomics to investigate the presence of antimicrobial resistance determinants and the genomic relatedness among strains isolated in the country [6, 16, 17]. Also, few studies have conducted worldwide investigations on the occurrence of antimicrobial efflux pump and heavy metal tolerance encoding genes in *S*. Infantis [18–22].

Therefore, the aims of this study were: (I) to assess the prevalence of antimicrobial efflux pump and heavy metal tolerance encoding genes, and (II) to evaluate the genomic relatedness of sequenced *S*. Infantis strains isolated in Brazil from various sources (food, the environment, humans and animals) from 2013 to 2018.

## 2. Material and methods

### 2.1. Bacterial genomes

A total of 80 whole-genome sequenced *S*. Infantis strains isolated from food (n = 27), farm and industry environments (n = 24), humans (n = 19), animals (n = 7) and animal feed (n = 3) sources were analyzed in this study. These strains were provided by the *Salmonella* reference laboratory collection of the Oswaldo Cruz Foundation of Rio de Janeiro (FIOCRUZ-RJ) and were isolated from 2013 to 2018, in states of the South (Santa Catarina, Rio Grande do Sul and Paraná), Southwest (São Paulo and Minas Gerais), Midwest (Mato Grosso do Sul and Goiás) and Northwest (Alagoas and Maranhão) regions of Brazil (Table 1).

**Table 1:** Strain identifiers and isolation data of the 80 *Salmonella* Infantis strains studied isolated from food (n = 27), farm and industry environments (n = 24), humans (n = 19), animals (n = 7) and animal feed (n = 3) between 2013 and 2018 in Brazil.

Strain identifiers and accession numbers			Isolation data		
Strain/Year	CFSAN no.	GenBank no.	Material	Source	State
SI 1348/13	CFSAN107127	AAWRHH000000000.1	Human feces	Human	PR
SI 2385/13	CFSAN107129	AAWRGU000000000.1	Soy	Food	PR
SI 2950/13	CFSAN107130	AAWRHS000000000.1	Human feces	Human	AL
SI 2951/13	CFSAN107131	AAWRHN000000000.1	Human feces	Human	AL
SI 3156/13	CFSAN107132	AAWRGH000000000.1	Disposable shoe cover	Environment	SC
SI 5025/13	CFSAN107133	AAWRGA000000000.1	Human feces	Human	SC
SI 124/14	CFSAN107134	AAWRDW000000000.1	Swine feces	Animal	RS
SI 210/14	CFSAN107136	AAWREM000000000.1	Dragging swab	Environment	SC
SI 212/14	CFSAN107137	AAWRDZ000000000.1	Dragging swab	Environment	SC
SI 388/14	CFSAN107138	AAWRER000000000.1	Soybean animal meal	Animal feed	SP
SI 583/14	CFSAN107139	AAWREP000000000.1	Chicken carcass	Food	SC
SI 584/14	CFSAN107140	AAWREX000000000.1	Pasta containing ham	Food	SC
SI 677/14	CFSAN107141	AAWRFG000000000.1	Carcass cleaning wipe	Food	SC
SI 723/14	CFSAN107142	AAWRFD000000000.1	Dragging swab	Environment	SC
SI 982/14	CFSAN107143	AAWRHV000000000.1	Chicken feces	Animal	RS
SI 1143/14	CFSAN107144	AAWRHU000000000.1	Chicken feces	Animal	RS
SI 1284/14	CFSAN107145	AAWRIM000000000.1	Dragging swab	Environment	SC
SI 1380/14	CFSAN107146	AAWRIF000000000.1	Chicken feces	Animal	RS
SI 1408/14	CFSAN107148	AAWRIL000000000.1	Human feces	Human	RS
SI 1409/14	CFSAN107149	AAWRHF000000000.1	Human feces	Human	RS
SI 1441/14	CFSAN107150	AAWRHL000000000.1	Mayonnaise	Food	RS
SI 1711/14	CFSAN107151	AAYKFJ000000000.1	Chicken feces	Animal	RS
SI 2378/14	CFSAN107152	AAWRHR000000000.1	Truck swab	Environment	SC
SI 2430/14	CFSAN107153	AAWRHO000000000.1	Mixed meat sausage	Food	SC
SI 2461/14	CFSAN107154	AAWRGI000000000.1	Chicken carcass	Food	SC
SI 2463/14	CFSAN107155	AAYKFK000000000.1	Chicken carcass	Food	SC
SI 2548/14	CFSAN107156	AAWRDS000000000.1	Chicken feces	Animal	RS
SI 3836/14	CFSAN107160	AAXBHC000000000.1	Dragging swab	Environment	RS
SI 4882/14	CFSAN107164	AAXBHW000000000.1	Chicken carcass	Food	MG
SI 4892/14	CFSAN107165	AAXAKM000000000.1	Chicken wings	Food	MG
SI 4895/14	CFSAN107166	AAXAKH000000000.1	Chicken carcass	Food	MG
SI 4901/14	CFSAN107167	AAXAKN000000000.1	Pig snout	Food	MG
SI 5247/14	CFSAN107168	AAXAKJ000000000.1	Chicken upper leg and thigh	Food	MG
SI 342/15	CFSAN107171	AAXHSY000000000.1	Swine heart	Food	SC
SI 444/15	CFSAN107172	AAXHRH000000000.1	Pork filet	Food	SC
SI 447/15	CFSAN107173	AAXHRI000000000.1	Smoked and salted pork meat	Food	SC
SI 1809/15	CFSAN107179	AAXHSE000000000.1	Meat animal meal	Animal feed	SC
SI 1816/15	CFSAN107180	AAXHVG000000000.1	Poultry viscera animal meal	Animal feed	SC
SI 2280/15	CFSAN107182	AAXHUK000000000.1	Chicken carcass	Food	SC
SI 2302/15	CFSAN107183	AAXHUC000000000.1	Cleaning wipe	Environment	SC
SI 2370/15	CFSAN107185	AAXHUH000000000.1	Carcass cleaning wipe	Food	SC
SI 2869/15	CFSAN107190	AAXHUP000000000.1	Chicken upper leg	Food	MG
SI 3056/15	CFSAN107193	AAXHUJ000000000.1	Chicken carcass	Food	MG
SI 4764/15	CFSAN107197	AAXHVH000000000.1	Cleaning wipe	Environment	SC
SI 5391/15	CFSAN107200	AAXHUD000000000.1	Disposable shoe cover	Environment	SC
SI 5837/15	CFSAN107201	AAXHTN000000000.1	Disposable shoe cover	Environment	SC
SI 5853/15	CFSAN107202	AAXJLL000000000.1	Disposable shoe cover	Environment	SC
SI 5859/15	CFSAN107203	AAXHWB000000000.1	Disposable shoe cover	Environment	SC
SI 5911/15	CFSAN107204	AAXHVK000000000.1	Cleaning wipe	Environment	SC
SI 5912/15	CFSAN107205	AAYKGL000000000.1	Cleaning wipe	Environment	SC
SI 5915/15	CFSAN107206	AAYKGJ000000000.1	Cleaning wipe	Environment	SC
SI 5923/15	CFSAN107207	AAYKGQ000000000.1	Cleaning wipe	Environment	SC
SI 220/16	CFSAN107212	AAYKGB000000000.1	Cleaning wipe	Environment	SC
SI 3687/16	CFSAN107222	AAYKGA000000000.1	Chicken carcass	Food	SC
SI 4447/16	CFSAN107224	AAYKGC000000000.1	Pork sausage	Food	SC
SI 5946/16	CFSAN107226	AAYAAA000000000.1	Pork rib	Food	SC
SI 6987/16	CFSAN107229	AAYAIC000000000.1	Human feces	Human	MA
SI 7876/16	CFSAN107233	AAYAFO000000000.1	Human feces	Human	RS
SI 11/17	CFSAN107235	AAYARD000000000.1	Dragging swab	Environment	PR
SI 23/17	CFSAN107237	AAYAFK000000000.1	Dragging swab	Environment	PR
SI 238/17	CFSAN107238	AAYAFN000000000.1	Dragging swab	Environment	PR
SI 872/17	CFSAN107239	AAYAFR000000000.1	Chicken carcass	Food	MG
SI 1171/17	CFSAN107242	AAYAFL000000000.1	Soil	Environment	SP
SI 1256/17	CFSAN107243	AAYAFP000000000.1	Soil	Environment	SP
SI 2580/17	CFSAN107259	AAYKFO000000000.1	Human feces	Human	SC
SI 2953/17	CFSAN107261	AAYKFZ000000000.1	Human fecal swab	Human	GO
SI 2954/17	CFSAN107262	AAYKFE000000000.1	Human fecal swab	Human	GO
SI 3380/17	CFSAN107263	AAYKFP000000000.1	Human fecal swab	Human	GO
SI 3877/17	CFSAN107264	AAYKFX000000000.1	Chicken wings	Food	MG
SI 3906/17	CFSAN107265	AAYKFS000000000.1	Sieve residue	Environment	SP
SI 4065/17	CFSAN107266	AAYKFR000000000.1	Human feces	Human	PR
SI 4067/17	CFSAN107267	AAYKGD000000000.1	Human feces	Human	PR
SI 4069/17	CFSAN107268	AAYKFD000000000.1	Human blood	Human	PR
SI 52/18	CFSAN107270	AAYKFI000000000.1	Chicken carcass	Food	MG
SI 331/18	CFSAN107273	AAYKFT000000000.1	Human fecal swab	Human	GO
SI 623/18	CFSAN107279	AAYKFY000000000.1	Human feces	Human	SC
SI 661/18	CFSAN107280	AAYKFW000000000.1	Human feces	Human	MS
SI 942/18	CFSAN107281	AAYKFM000000000.1	Human fecal swab	Human	RS
SI 1634/18	CFSAN107284	AAYKFQ000000000.1	Yellowtail amberjack fish meat	Food	SC
SI 2676/18	CFSAN107285	AAYKFF000000000.1	Avian reproductive matrix	Animal	GO

Cleaning wipe: similar to synthetic tissues for domestic cleaning sold commercially; employed in the isolation procedure of microorganisms from surfaces in Brazil.

AL, Alagoas; BA, Bahia; GO, Goiás; MA, Maranhão; MG, Minas Gerais; MS, Mato Grosso do Sul; PE, Pernambuco; PR, Paraná; RS, Rio Grande do Sul; SC, Santa Catarina; SP, São Paulo.

These data are available in details at Vilela et al. 2021 [23].

The extraction of the genomic DNA was performed by the phenol-chloroform-isoamyl alcohol method, as previously described [23], and 1ng of the extracted DNA was used for the preparation of libraries with the Nextera XT DNA kit (Illumina, San Diego, CA). Genomes were sequenced in the Illumina MiSeq platform using the 2 X 150-bp paired-end MiSeq Reagent Kit version 3 (Illumina, San Diego, CA). Genome drafts were assembled with SKESA 2.2. Quality control was performed in the MicroRunQC workflow.

The complete isolation data, accession numbers and metadata of the genomic sequences of the 80 *S*. Infantis strains analyzed have been published in Vilela and collaborators [23] and are also partially displayed in Table 1.

### 2.2. Search of efflux pump coding genes

The Resistance Gene Identifier (RGI) tool of the Comprehensive Antibiotic Resistance Database (CARD; https://card.mcmaster.ca/analyze/rgi) [24] was used to search for resistance genes responsible for the coding of antimicrobial efflux pumps for each of the 80 sequenced *S*. Infantis strains studied (Table 1). Default parameters were applied in the analysis. Only genes related to antimicrobial efflux and showing ≥80% identity/length were included in the results.

### 2.3. Search of heavy metal tolerance genes

The “Stress genotypes” filter, one of the automatic features of the Isolate Browser of NCBI’s Pathogen Detection database utilizing AMRFinderPlus curated database (https://www.ncbi.nlm.nih.gov/pathogens/isolates/), was used to detect heavy metal tolerance genes in each of the 80 sequenced *S*. Infantis studied (Table 1). Only genes related to heavy metal tolerance were included in the results.

### 2.4. Phylogenetic analysis

The genomic relatedness of the 80 sequenced *S*. Infantis strains was accessed by core genome Multi-locus Sequence Typing (cgMLST) and was performed from a set of reads in the cgMLSTFinder 1.1 tool (available at https://cge.cbs.dtu.dk/services/cgMLSTFinder/) using the *Salmonella* (Enterobase) filter [25]. The complete genome of the chicken isolate SINFA (Genbank accession number LN649235.1), isolated in the United Kingdom in 1973, also was included for comparison purposes.

Two different subsets were phylogenetically analyzed. The first analysis included the 80 *S*. Brazilian strains to provide an overview of the genomes studied in relation to the efflux pump and heavy metal tolerance genes found. In the second analysis, the 80 strains were analyzed in combination with 40 additional *S*. Infantis genomes from eight countries to provide a more global view of the genomes studied. These 40 genomes were selected in NCBI’s Pathogen Detection and were selected from strains of diverse years and sources including isolates from Canada, Ecuador, Germany, Mexico, Peru, South Africa, United Kingdom and the United States. Detailed descriptions and accession numbers of the 40 additional *S*. Infantis genomes are displayed in S2 Table.

### 2.5. Statistical analysis

The results of the search for antimicrobial efflux pump and heavy metal tolerance encoding genes were expressed in percentages. The Chi-square test was employed using the software GraphPad Prism 5 (GraphPad Software, San Diego, CA, USA) in order to verify possible associations between specific profiles of interest among the antimicrobial efflux pumps and heavy metal tolerance genes found among the strains studied.

## 3. Results

### 3.1. Antimicrobial efflux pump encoding genes

A total of 20 genes have been detected, with 17 of these genes harbored by 100% of the 80 *S*. Infantis strains studied (Table 2; S1 Table) including genes *acrA*, *acrB*, *baeR*, *crp*, *emrB*, *emrR*, *hns*, *kdpE*, *kpnF*, *marA*, *marR*, *mdtK*, *msbA*, *rsmA*, *sdiA*, *soxR* and *soxS*. Other genes were variably observed including *golS* in 79 strains (98.75%), *mdfA* in 47 strains (58.75%), and *tet(A)* in 30 strains (37.5%). According to CARD, these genes are able to promote resistance to one up to sixteen classes of antibiotics (aminocoumarins, aminoglycosides, carbapenems, cephalosporins, cephamycins, diaminopyrimidine compounds, glycylcyclines, monobactams, penams, penems, phenicols, quinolones, rifamycins, tetracyclines, macrolides and nitroimidazoles), biocides (benzalkonium chloride and triclosan), dyes (rhodamine) and antibiotic peptides (S3 Table). The combination of multiple genes detected among the strains studied resulted in four different efflux pump encoding gene profiles, that are displayed in Table 2.

**Table 2:** Antimicrobial efflux pump encoding gene (ARG) profiles detected among the 80 *Salmonella* Infantis strains studied isolated from food (n = 27), farm and industry environments (n = 24), humans (n = 19), animals (n = 7) and animal feed (n = 3) in Brazil between 2013 and 2018.

Profile no.	Gene profiles	Isolation sources					Total
		FO	EN	HU	AN	AF	
1	*acrA*, *acrB*, *baeR*, *crp*, *emrB*, *emrR*, *golS*, *hns*, *kdpE*, *kpnF*, *marA*, *marR*, *mdfA*, *mdtK*, *msbA*, *rsmA*, *sdiA*, *soxR*, *soxS*	14	11	14	6	1	46
2	*acrA*, *acrB*, *baeR*, *crp*, *emrB*, *emrR*, *golS*, *hns*, *kdpE*, *kpnF*, *marA*, *marR*, *mdtK*, *msbA*, *rsmA*, *sdiA*, *soxR*, *soxS*, *tet(A)*	12	11	4	1	2	30
3	*acrA*, *acrB*, *baeR*, *crp*, *emrB*, *emrR*, *golS*, *hns*, *kdpE*, *kpnF*, *marA*, *marR*, *mdtK*, *msbA*, *rsmA*, *sdiA*, *soxR*, *soxS*	1	2	-	-	-	3
4	*acrA*, *acrB*, *baeR*, *crp*, *emrB*, *emrR*, *hns*, *kdpE*, *kpnF*, *marA*, *marR*, *mdfA*, *mdtK*, *msbA*, *rsmA*, *sdiA*, *soxR*, *soxS*	-	-	1	-	-	1

FO, food; EN, environment; HU, human; AN, animal; AF; animal feed.

The complete distribution of the genes in all 80 strains is demonstrated in S2 Table and the specific spectrum of drug resistance promoted by each of the 20 genes detected is shown in S3 Table.

### 3.2. Heavy metal tolerance encoding genes

All the 80 *S*. Infantis strains studied (100%) harbored the *arsR* gene, related to arsenic tolerance. A total of 79 strains (98.75%) harbored *golS* and *golT*, related to gold tolerance. A complete *sil* operon (*silABCDEFPRS*), related to silver tolerance, was detected in 29 strains (36.25%), while a unique strain (1.25%) harbored only *silE* gene. A single strain (1.25%) also harbored genes *merR* and *merT*, related to mercury tolerance. The distribution of the genes related to arsenic, gold, silver and mercury tolerance are displayed for each of the 80 *S*. Infantis strains analyzed in S2 Table.

### 3.3. Phylogenetic analyses

**Fig 1:**
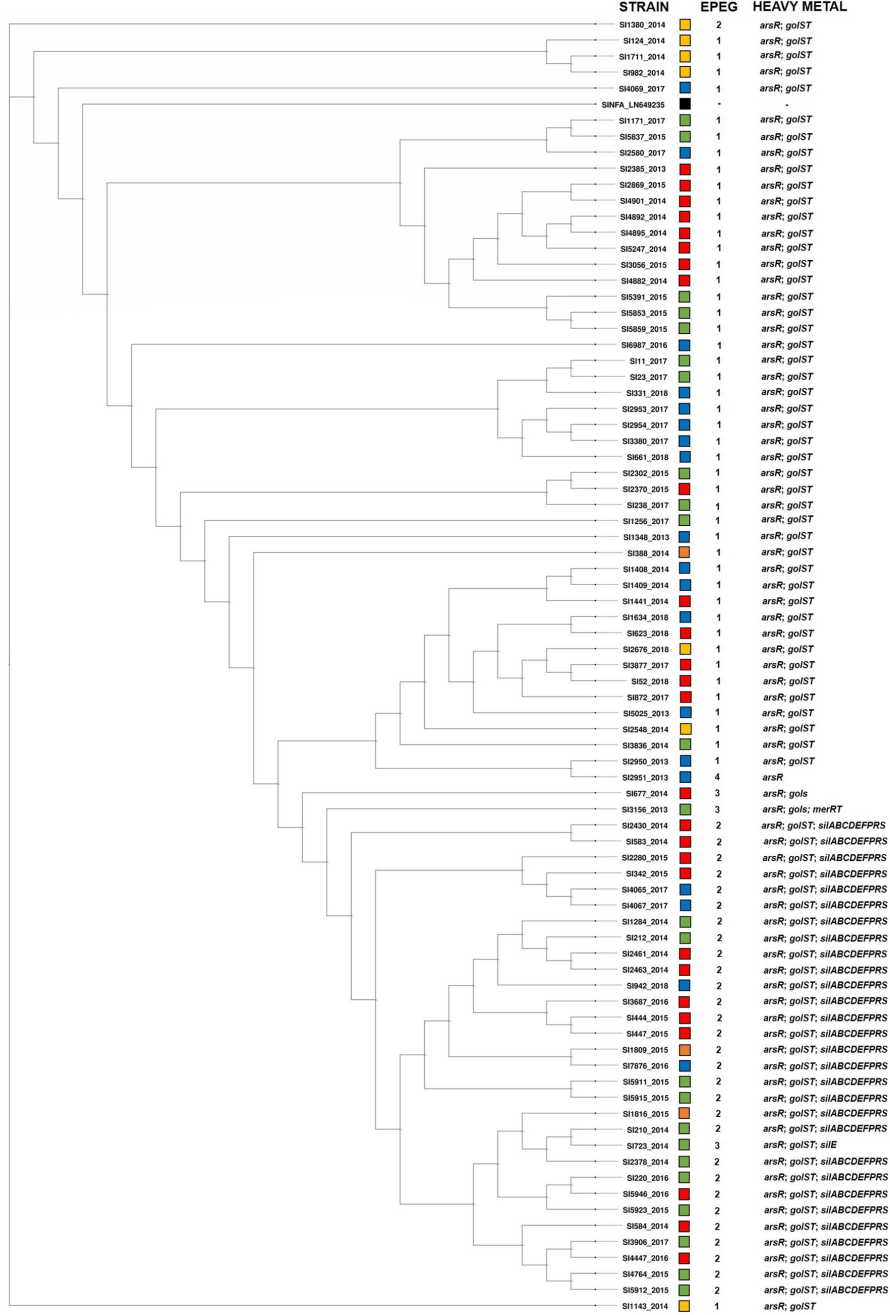
Phylogenetic tree based on the core-genome multi-locus sequence typing (cgMLST) analysis of the 80 whole-genome sequenced *S*. Infantis strains studied, isolated from food (red squares; n = 27), farm and industry environments (green squares; n = 24), humans (blue squares; n = 19), animals (yellow squares; n = 7) and animal feed (orange squares; n = 3) between 2013 and 2018 in Brazil. *S*. Infantis reference strain SINFA LN649235.1 (black square) was included for comparison purposes. Additional information regarding the isolation sources, efflux pump encoding genes (EPEG) and heavy metal tolerance genes are included. Profile 1 (*acrA*, *acrB*, *baeR*, *crp*, *emrB*, *emrR*, *golS*, *hns*, *kdpE*, *kpnF*, *marA*, *marR*, *mdfA*, *mdtK*, *msbA*, *rsmA*, *sdiA*, *soxR*, *soxS*); Profile 2 (*acrA*, *acrB*, *baeR*, *crp*, *emrB*, *emrR*, *golS*, *hns*, *kdpE*, *kpnF*, *marA*, *marR*, *mdtK*, *msbA*, *rsmA*, *sdiA*, *soxR*, *soxS*, *tet(A)*); Profile 3 (*acrA*, *acrB*, *baeR*, *crp*, *emrB*, *emrR*, *golS*, *hns*, *kdpE*, *kpnF*, *marA*, *marR*, *mdtK*, *msbA*, *rsmA*, *sdiA*, *soxR*, *soxS*); Profile 4 (*acrA*, *acrB*, *baeR*, *crp*, *emrB*, *emrR*, *hns*, *kdpE*, *kpnF*, *marA*, *marR*, *mdfA*, *mdtK*, *msbA*, *rsmA*, *sdiA*, *soxR*, *soxS*).

**Fig 2:**
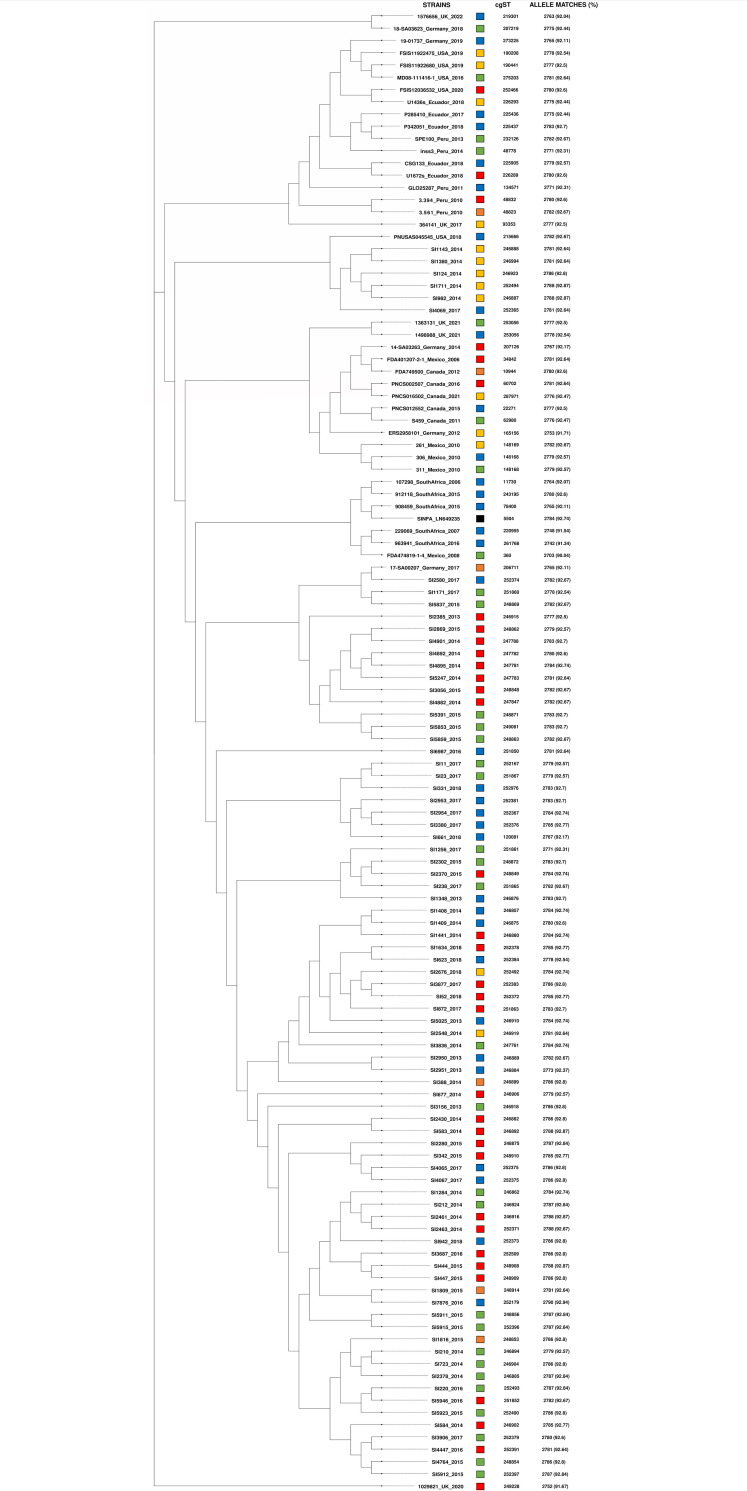
Phylogenetic tree based on the core-genome multi-locus sequence typing (cgMLST) analysis of the 120 genomes of *S*. Infantis strains from Brazil, Canada, Ecuador Germany, Mexico, Peru, South Africa, United Kingdom (UK) and United States (US). The 120 strains were isolated from food (red squares), environments (green squares), humans (blue squares), animals (yellow squares) and animal feed (orange squares). *S*. Infantis reference strain SINFA LN649235.1 (black square) was included for comparison purposes. Additional information regarding the isolation sources, core-genome sequence types (cgSTs) and the number and percentages of the identified alleles are included.

The phylogenetic trees based on the cgMLST analyses of 3,002 genes common to the *Salmonella* genus are presented in Figs 1 and 2. In Fig 1, the 80 *S*. Infantis strains are displayed alongside the profiles of efflux pump (Table 2) and heavy metal tolerance encoding genes. In Fig 2, the 120 *S*. Infantis strains are displayed along with the core genome sequence types (cgSTs) identified and numbers and percentages of the matched alleles found.

Regarding the analysis in Fig 1, it was possible to observe a strong genetic association also demonstrated statistically (*p* < .00001) among 46 strains harboring *golS*, *golT*, *arsR* and the profile 1 of efflux pump encoding genes, as well as 29 strains harboring *golS*, *golT*, *arsR*, the *sil* operon and the profile 2 of efflux pump encoding genes (Fig 1; Table 2; S1 Fig), regardless of their isolation sources, materials and locations.

As illustrated in Fig 2, an extensive variation of cgSTs was observed among both Brazilian and international *S*. Infantis genomes (Fig 2). Of the 120 genomes analyzed, only six shared the same cgSTs. Two Brazilian strains isolated in 2017 from humans (SI4065 and SI4067) belonged to cgST 252375. Two genomes of human and environmental sources isolated in 2010 in Mexico (306 and 311) were assigned to cgST 148168. Finally, two genomes of human and environmental sources isolated in 2021 from the United Kingdom belonged to cgST 253056 (Fig 2).

## 4. Discussion

The increasing rates of drug-resistant NTS have become a public health and food safety concern worldwide [2, 3]. *S*. Infantis is a major ubiquitous NTS serovar, present in food, the environment, humans and animal sources, and also associated with increasing resistant rates to antimicrobial compounds of human and veterinary use [4–6, 16]. Previously, the 80 *S*. Infantis strains studied were analyzed using ResFinder (Center for Genomic Epidemiology) and AMRFinder (Pathogen Detection—NCBI) for resistance gene detection [6], where acquired resistance genes conferring resistance to β-lactams, diaminopyrimidine compounds, amphenicols, aminoglycosides, tetracycline, sulfonamide, and chromosomal point mutations associated to quinolone and antimicrobial peptide resistance, have been detected (S2 Table).

Antimicrobial efflux pumps have been described as important mechanisms of antimicrobial resistance among Gram-negative bacteria [8, 9]. However, despite the clinical and veterinary importance and the increasing resistance rates, there is little information about the frequency and diversity of antimicrobial efflux pump encoding genes in *S*. Infantis. According to the current published literature, only one study has evaluated the genetic variability among the sequences of *acr*, *mar* and *sox* genes in *S*. Infantis mutants and its correlation to quinolone resistance [18].

In the present study, the *S*. Infantis strains analyzed harbored 20 different types of efflux pumps encoding genes using the CARD tool that are capable to promote resistance from one to sixteen classes of antibiotics, biocides, dyes and antibiotic peptides (S3 Table). Previously, ResFinder and AMRFinder were also employed to determine the genotypic resistance profile of the same set of strains, and were able to identify the efflux pump encoding genes *msdA*, *mdsB*, *tet(A)* and point mutations in *acrB*. Interestingly, both *acrA* and *tet(A)* were detected at the same frequencies (S2 Table) as found herein [6]. Therefore, the results here obtained in comparison to data previously generated by other analysis tools suggested that differences in gene content may occur from platform to platform, and it possibly may be due to differences in the gene content deposited in their databases by their curators.

The *acrA* and *acrB* genes partially encode the well-studied AcrAB-TolC tripartite efflux system, which has been demonstrated to confer significant resistance to several antibiotic classes in NTS serovars [26]. However, the necessity of an intact set of genes in the efflux system’s and the possible occurrence of mutations may influence the resistance levels promoted [29]. In addition, the genes *marAR* and *soxRS* have been demonstrated to act in AcrAB-TolC system’s regulation and to be essential to its multidrug resistance action [27]. The *ermB* and *ermR* genes act in the promotion of increased quinolone resistance, mainly related to nalidixic acid, but its conjunct action with chromosomal point mutations and plasmid-borne genes can also favor the development of high resistance levels to fluoroquinolone drugs, such as ciprofloxacin [28]. The *mdfA* and *mdtK* genes have been reported and characterized to encode expressive resistance to tetracycline, chloramphenicol, norfloxacin, doxorubicin, acriflavine and biocides in the NTS serovar *S*. Typhimurium [29]. The *kpnF* gene, initially described in *Klebsiella pneumoniae*, also promotes significant resistance to several classes of antibiotics, antiseptics and disinfectants [30]. Finally, according to the CARD database, it is also important to mention genes like *baeR*, *crp*, *golS*, *hns*, *rsmA* and *sdiA*, which do not encode specific efflux pumps, but act as important regulators of *acr*, *erm* and *mdt* efflux systems.

In this way, this diverse occurrence of efflux pump encoding genes with broad spectrum of resistance found in high frequencies in *S*. Infantis strains may be a concern. The presence of these genes, in association to other genetic resistance determinants, could influence the resistance to drugs-of-choice for the treatment of human salmonellosis (such as fluoroquinolones and cephalosporins) [31, 32] and also to drugs less employed in human therapy but broadly employed in the veterinary area (such as tetracycline, phenicols and aminoglycosides) [33]. Additionally, the presence of genes able to promote resistance to non-antibiotic compounds demonstrate that antiseptics, disinfectants and related products could also show some inefficacy against *S*. Infantis, facilitating a possible co-selection of antibiotic-resistant strains.

The wide presence of heavy metal tolerance genes in bacteria has been described as a result of the selective pressure due to the combination of the occurrence of these compounds in nature, environmental pollution, and its applications in medical devices, disinfectants, antiseptics and preservatives [10–12]. In the present study, arsenic (*arsR*), gold (*golS* and *golT*), silver (*silABCDEFPRS*) and mercury (*merR* and *merT*) tolerance genes were detected among the 80 *S*. Infantis strains analyzed.

The *sil* operon (*silABCDEFPRS*) encodes the formation of the SilE periplasmic silver binding-protein, SilP and SilABC silver efflux pumps, SilF and SilG chaperones and SilS and SilR two-component signal transduction pair [11, 12, 34]. The first detailed characterization of the *sil* operon was provided after a major hospital outbreak with high mortality rates among burned patients caused by a broadly resistant *S*. Typhimurium clone [35], and since then, these genes have been reported in a wide range of clinical and environmental bacteria [11, 34]. Gold tolerance genes *golS* and *golT* are described in NCBI’s Pathogen Detection Gene Catalog (https://www.ncbi.nlm.nih.gov/pathogens/refgene/) to encode a sensor transcriptional regulator and a gold translocating P-type ATPase, respectively. The arsenic tolerance gene *arsR* encodes a responsive trans-acting transcriptional repressor and is part of the minimum arsenic tolerance operon *aRBC* [11, 12], whose genes have been reported previously among *S*. Infantis strains [22]. Related to regulatory and transport functions, the mercury tolerance genes *merR* and *merT* belong to the *mer* operon [11, 12], and have been reported among *S*. Infantis strains from several countries in association to the pESI mega plasmid [19–21].

Silver, gold and mercury compounds have been explored over the years in diverse applications for human medicine due to their high toxicity for bacteria [12, 36]. For example, silver can be found in the coating of medical devices like catheters and endotracheal tubes, as part of dental amalgam, and used for the treatment of wounds and prophylaxis of gonococcal ophthalmia neonatorum [12]. Gold has been employed in medical imaging devices, in the treatment of human diseases, and more recently, in the development of nanoparticles, including those with antimicrobial potential [12, 36]. In the environmental and veterinary field, silver has been applied for water treatment, while organic arsenic compounds have been employed as pesticides and feed supplements for food-producing animals [11]. Finally, despite its toxicity, mercury has also been used in agriculture and in human medicine in the form of organic and inorganic compounds, respectively [11, 12].

It is important to mention that the acquisition and transference of resistance genes in bacteria have been largely associated to their presence in plasmids [21, 22]. However, as demonstrated previously [6], the 80 *S*. Infantis strains studied have a low frequency and diversity of plasmids. These data, when compared with the profiles of efflux pump and heavy metal tolerance encoding genes here detected (S2 Table) suggest that these genes may have a chromosomal location in the strains analyzed. For example, as mentioned above, mercury tolerance genes *merR* and *merT* are highly associated to *S*. Infantis pESI endemic plasmid [19–21]. However, this correlation was not observed in this study (S2 Table), since the strain showing this gene profile has been previously demonstrated not to carry this type of plasmid [6].

Therefore, the presence of genes conferring tolerance to heavy metals employed in the medical, veterinary and environmental field among *S*. Infantis strains may also warn us on the necessity of increased surveillance of these genetic traits, due to their potential role in the development of resistance against these non-antibiotic compounds and the co-selection of drug-resistance strains of this serovar.

It should also be addressed that additional future research in the field, such as targeted gene expression and phenotypic analyses, could greatly contribute to the elucidation of the role and presumed connections of the efflux pumps and heavy metal tolerance encoding genes here detected with antimicrobial resistance in *S*. Infantis.

Over the recent years, phylogenetic analyses based on genomic data have demonstrated their importance to epidemiology and tracking of antimicrobial resistance in pathogens of public health relevance [13, 14]. Among the various methods currently available for phylogenetic analyses, cgMLST is based in the allelic variations between the set of 3,002 conserved genes of the *Salmonella* genus [25], and has been developed and employed as an evolution of the legacy MLST, based in the allelic differences of seven housekeeping genes. While cgMLST has still been less employed in the study of *S*. Infantis strains [21, 37, 38], MLST has been largely employed over the years and shows a high global predominance of the sequence type ST32 for strains of this serovar [6, 16, 21, 22]. Previously, the dominance of ST32 has also been determined by MLST in the 80 Brazilian *S*. Infantis strains. In addition, pulsed-field gel electrophoresis (PFGE) showed the presence of three distinct clusters with a high overall similarity above 78%, and the single-nucleotide polymorphism (SNP) analysis of NCBI’s Pathogen Detection assigned the strains to 13 distinct SNP clusters, where seven of these also comprised international isolates and six were composed exclusively of Brazilian isolates [6].

Our Fig 1 did not demonstrate any clear distinction among the strains analyzed regarding their diverse isolation years, sources, materials or Brazilian states. However, some strains harbored combinations of specific profiles of heavy metal tolerance and efflux pump encoding genes were closely grouped in the phylogenetic tree and possessed a strong statistically significant association (Table 2; Fig 1; S1 Fig). Moreover, the results from Fig 2 revealed that the majority of the Brazilian strains possessed a significant proximity among each other and, in contrast, were clearly distinct from international isolates. Only five strains isolated from animals in 2014 (SI124, SI982, SI1143, SI1380, SI1711) and one from human in 2017 (SI4069) were closely located among international genomes (Fig 2).

Furthermore, based on the findings of cgMLST, an extensive diversity of cgSTs was recorded among both Brazilian and international *S*. Infantis genomes. It is interesting to note that previous studies also performed cgMLST to subtype *S*. Infantis isolated in European countries, and similarly reported the same high occurrence of MLST’s ST32 in contrast to a high diversity of cgSTs found by using cgMLST in related strains [21, 37, 38].

The results of the present study demonstrate that although, the *S*. Infantis strains studied isolated in Brazil may present a high overall genomic relatedness and a possible common occurrence in diverse sources, it was also possible to identify distinct strain subtypes in comparison to strains from other countries, as well as the presence of subgroups of strains showing different profiles for heavy metal tolerance and efflux pump encoding genes.

In conclusion, the high prevalence of diverse efflux pump encoding genes, the high occurrence of some heavy metal tolerance genes and the high genomic relatedness of *S*. Infantis strains sharing equal resistant profiles, regardless of their origin, warn us of the importance of strong surveillance measures to monitor resistance and possible transmission of strains of this serovar among diverse sources in Brazil. Together, these results provided new insights on the genomic diversity of *S*. Infantis strains circulating in Brazil using WGS.

## Supporting information

S1 Table Metadata of the additional 40 *Salmonella* Infantis genomes from diverse countries recovered from NCBI’s Pathogen Detection platform and included in the core genome multi-locus sequence typing (cgMLST) analysis. (PDF)

S2 Table Antimicrobial resistance and plasmid profiles of the 80 *Salmonella* Infantis strains studied isolated from food (n = 27), the environment (n = 24), humans (n = 19), animals (n = 7) and animal ration (n = 3) between 2013 and 2018 in Brazil. (PDF)

S3 Table List of the drug classes and resistance mechanisms as referred by the Comprehensive Antibiotic Resistance Database (CARD) of the efflux pump encoding genes detected among the 80 sequenced *Salmonella* Infantis strains studied isolated from food (n = 27), farm and industry environments (n = 24), humans (n = 19), animals (n = 7) and animal feed (n = 3) in Brazil between 2013 and 2018. (PDF)

S1 Fig Statistical analysis based on the Chi-Square test to verify the association among the 46 S. Infantis strains co-harboring *golS*, *golT*, *arsR* and the profile 1 of efflux pump encoding genes and 29 strains harboring *golS*, *golT*, *arsR*, the *sil* operon and the profile 2 of efflux pump encoding genes. (PDF)
