# Supplementary material for: Prevalence of efflux pump and heavy metal tolerance encoding genes among Salmonella enterica serovar Infantis strains from diverse sources in Brazil
Source: PLoS One. 2022 Nov 22;17(11):e0277979. doi: 10.1371/journal.pone.0277979 (PMC9681071; doi:10.1371/journal.pone.0277979)
Supplement: S1 Table — (PDF) [file pone.0277979.s001.pdf]

Table S1 - Metadata of the additional 40 *Salmonella* Infantis genomes from diverse countries recovered from NCBI's Pathogen Detection platform and included in the core genome multi-locus sequence typing (cgMLST) analysis.

Strain	Collection date	Location	Isolation source	Isolation type	BioSample	Assembly	Isolate
PNUSAS045545	2018	USA	Stool	Human	SAMN09791483	GCA_008458465.1	PDT000360932.1
FSIS11922475	2019	USA	Young chicken	Animal	SAMN12213724	GCA_007801115.1	PDT000538820.1
FSIS11922680	2019	USA	Young chicken	Animal	SAMN12228446	GCA_007918635.1	PDT000539773.1
FSIS12036532	2020	USA	Comminuted chicken	Food	SAMN17175485	GCA_016436305.1	PDT000925642.1
MD08-111416-1	2016	USA	Water	Environment	SAMN19856631	GCA_019036465.1	PDT001076716.1
1363131	2021	United Kingdom	Environment	Environment	SAMN21389114	GCA_019938715.1	PDT001127307.1
1498988	2021	United Kingdom	Human	Human	SAMN22575510	GCA_020650535.1	PDT001162426.1
364141	2017	United Kingdom	Animal	Animal	SAMN07812230	GCA_009560875.1	PDT000253765.2
1029821	2020	United Kingdom	Food	Food	SAMN16849643	GCA_015652285.1	PDT000894823.1
1576656	2022	United Kingdom	Human	Human	SAMN25891193	GCA_022222205.1	PDT001249079.1
PNCS012552	2015	Canada	Stool	Human	SAMN26791278	GCA_022900555.1	PDT001283160.1
PNCS002507	2016	Canada	Frozen breaded chicken	Food	SAMN18211016	GCA_019133875.1	PDT001082071.1
PNCS016502	2021	Canada	Pork	Animal	SAMN22039197	GCA_020398345.1	PDT001146933.1
FDA749500	2012	Canada	Canola meal	Animal feed	SAMN02918921	GCA_008618295.1	PDT000087535.2
S459	2011	Canada	Soil	Environment	SAMN20150139	GCA_020672185.1	PDT001164982.1
17-SA00207	2017	Germany	Feed	Animal feed	SAMEA6057688	GCA_015044155.1	PDT000606531.1
14-SA03263	2014	Germany	Food	Food	SAMEA6058158	GCA_010301525.1	PDT000606987.1
18-SA03623	2018	Germany	Environment	Environment	SAMEA6058273	GCA_009618835.1	PDT000607101.1
19-01737	2019	Germany	Stool	Human	SAMEA7540893	GCA_020159065.1	PDT001137379.1
ERS2958101	2012	Germany	Animal	Animal	SAMEA5150359	GCA_022393355.1	PDT001254168.1
3.394	2010	Peru	Meat	Food	SAMN05417413	GCA_005967375.1	PDT000140607.2
3.561	2010	Peru	Animal feed	Animal feed	SAMN05417416	GCA_006030655.1	PDT000140640.2

Strain	Collection date	Location	Isolation source	Isolation type	BioSample	Assembly	Isolate
GLO25287	2011	Peru	Biological fluid/tissue	Human	SAMN07244646	GCA_004189575.1	PDT000338756.1
SPE100	2013	Peru	Hospital	Environment	SAMN10837491	GCA_012223825.1	PDT000721023.1
inss3	2014	Peru	Clinic	Environment	SAMN10722782	GCA_012939845.1	PDT000730276.2
107298	2006	South Africa	Blood	Human	SAMEA11580049	GCA_023491965.1	PDT001310299.1
912118	2015	South Africa	Wound	Human	SAMEA11580307	GCA_023498145.1	PDT001310349.1
229069	2007	South Africa	Rectal swab	Human	SAMEA11580055	GCA_023491625.1	PDT001310326.1
963941	2016	South Africa	Stool	Human	SAMEA11580244	GCA_023486235.1	PDT001310369.1
908459	2015	South Africa	Urine	Human	SAMEA11580212	GCA_023498165.1	PDT001310347.1
311	2010	Mexico	River water	Environment	SAMN10261431	GCA_005553365.1	PDT000395947.1
306	2010	Mexico	Stool	Human	SAMN10261426	GCA_005698475.1	PDT000395950.1
FDA474819-1-4	2008	Mexico	Environmental	Environment	SAMN02846130	GCA_008915745.2	PDT000146150.3
FDA401207 2-1	2006	Mexico	Chocolate candy piece	Food	SAMN02845864	GCA_008115505.1	PDT000066975.2
261	2010	Mexico	Cow stool	Animal	SAMN10261399	GCA_005795595.1	PDT000395884.1
CSG133	2018	Ecuador	Human	Human	SAMEA7302380	GCA_019813995.1	PDT001119464.1
U1436s	2018	Ecuador	Caecal sample	Animal	SAMEA7302493	GCA_020867305.1	PDT001173953.1
U1672s	2018	Ecuador	Chicken carcasses	Food	SAMEA7302507	GCA_020867165.1	PDT001173959.1
P342051	2018	Ecuador	Urine	Human	SAMN24594576	GCA_021395095.1	PDT001219601.1
P285410	2017	Ecuador	Skin and soft tissue	Human	SAMN24594573	GCA_021394515.1	PDT001219599.1
