# Supplementary material for: Prevalence of efflux pump and heavy metal tolerance encoding genes among Salmonella enterica serovar Infantis strains from diverse sources in Brazil
Source: PLoS One. 2022 Nov 22;17(11):e0277979. doi: 10.1371/journal.pone.0277979 (PMC9681071; doi:10.1371/journal.pone.0277979)
Supplement: S2 Table — (PDF) [file pone.0277979.s002.pdf]

Table S2 - Antimicrobial resistance and plasmid profiles of the 80 *Salmonella* Infantis strains studied isolated from food (n=27), the environment (n=24), humans (n=19), animals (n=7) and animal ration (n=3) between 2013 and 2018 in Brazil.

Strain no.	Efflux pump encoding genes	Heavy metal tolerance genes	Acquired resistance genes*	Chromosomal point mutations*	Plasmids*
SI 1348/13	*acrA, acrB, baeR, crp, emrB, emrR, golS, hns, kdpE, kpnF, marA, marR, mdfA, mdtK, msbA, rsmA, sdiA, soxR, soxS*	*arsR, golST*	*aac(6')-Iaa, mdsA, mdsB*	*gyrB* (Gln624→Lys), *parC* (Thr57→Ser and Thr255→Ser), *acrB* (Phe28→Leu and Leu40→Pro)	-
SI 2385/13	*acrA, acrB, baeR, crp, emrB, emrR, golS, hns, kdpE, kpnF, marA, marR, mdfA, mdtK, msbA, rsmA, sdiA, soxR, soxS*	*arsR, golST*	*aac(6')-Iaa, mdsA, mdsB*	*gyrB* (Gln624→Lys), *parC* (Thr57→Ser and Thr255→Ser), *acrB* (Phe28→Leu and Leu40→Pro)	-
SI 2950/13	*acrA, acrB, baeR, crp, emrB, emrR, golS, hns, kdpE, kpnF, marA, marR, mdfA, mdtK, msbA, rsmA, sdiA, soxR, soxS*	*arsR, golST*	*aac(6')-Iaa, mdsA, mdsB*	*gyrB* (Gln624→Lys), *parC* (Thr57→Ser and Thr255→Ser), *acrB* (Phe28→Leu and Leu40→Pro)	-
SI 2951/13	*acrA, acrB, baeR, crp, emrB, emrR, golS, hns, kdpE, kpnF, marA, marR, mdtK, msbA, rsmA, sdiA, soxR, soxS*	*arsR*	*aac(6')-Iaa*	*gyrB* (Gln624→Lys), *parC* (Thr57→Ser and Thr255→Ser), *acrB* (Phe28→Leu and Leu40→Pro)	-
SI 3156/13	*acrA, acrB, baeR, crp, emrB, emrR, hns, kdpE, kpnF, marA, marR, mdfA, mdtK, msbA, rsmA, sdiA, soxR, soxS*	*arsR, golST, merRT*	*aac(6')-Iaa, aadA12,* $bla_{\text{TEM-1}}$*, mdsA, mdsB*	*gyrB* (Gln624→Lys), *parC* (Thr57→Ser and Thr255→Ser), *acrB* (Phe28→Leu and Leu40→Pro)	IncX1
SI 5025/13	*acrA, acrB, baeR, crp, emrB, emrR, golS, hns, kdpE, kpnF, marA, marR, mdfA, mdtK, msbA, rsmA, sdiA, soxR, soxS*	*arsR, golST*	*aac(6')-Iaa, mdsA, mdsB*	*gyrB* (Gln624→Lys), *parC* (Thr57→Ser and Thr255→Ser), *acrB* (Phe28→Leu and Leu40→Pro)	-
SI 124/14	*acrA, acrB, baeR, crp, emrB, emrR, golS, hns, kdpE, kpnF, marA, marR, mdfA, mdtK, msbA, rsmA, sdiA, soxR, soxS*	*arsR, golST*	*aac(6')-Iaa,* $bla_{\text{CMY-2}}$*, mdsA, mdsB*	*gyrB* (Gln624→Lys), *parC* (Thr57→Ser and Thr255→Ser), *acrB* (Phe28→Leu and Leu40→Pro), *pmrA* (Asp28-Tyr)	IncI1-I (Alpha)
SI 210/14	*acrA, acrB, baeR, crp, emrB, emrR, golS, hns, kdpE, kpnF, marA, marR, mdtK, msbA, rsmA, sdiA, soxR, soxS, tet(A)*	*arsS, golST, silABCDEFPRS*	*aac(6')-Iaa,* $bla_{\text{TEM-1}}$*, floR, dfrA8, tet(A), mdsA, mdsB*	*gyrB* (Gln624→Lys), *parC* (Thr57→Ser and Thr255→Ser), *acrB* (Phe28→Leu and Leu40→Pro)	-
SI 212/14	*acrA, acrB, baeR, crp, emrB, emrR, golS, hns, kdpE, kpnF, marA, marR, mdtK, msbA, rsmA, sdiA, soxR, soxS, tet(A)*	*arsS, golST, silABCDEFPRS*	*aac(6')-Iaa, aadA12,* $bla_{\text{TEM-1}}$*, floR, dfrA8, tet(A), mdsA, mdsB*	*gyrB* (Gln624→Lys), *parC* (Thr57→Ser and Thr255→Ser), *acrB* (Phe28→Leu and Leu40→Pro)	IncX1
SI 388/14	*acrA, acrB, baeR, crp, emrB, emrR, golS, hns, kdpE, kpnF, marA, marR, mdfA, mdtK, msbA, rsmA, sdiA, soxR, soxS*	*arsR, golST*	*aac(6')-Iaa, mdsA, mdsB*	*gyrB* (Gln624→Lys), *parC* (Thr57→Ser and Thr255→Ser), *acrB* (Phe28→Leu and Leu40→Pro)	-
SI 583/14	*acrA, acrB, baeR, crp, emrB, emrR, golS, hns, kdpE, kpnF, marA, marR, mdtK, msbA, rsmA, sdiA, soxR, soxS, tet(A)*	*arsS, golST, silABCDEFPRS*	*aac(6')-Iaa,* $bla_{\text{TEM-1}}$*, floR, dfrA8, tet(A), mdsA, mdsB*	*gyrB* (Gln624→Lys), *parC* (Thr57→Ser and Thr255→Ser), *acrB* (Phe28→Leu and Leu40→Pro)	-

Strain no.	Efflux pump encoding genes	Heavy metal tolerance genes	Acquired resistance genes*	Chromosomal point mutations*	Plasmids*
SI 584/14	*acrA, acrB, baeR, crp, emrB, emrR, golS, hns, kdpE, kpnF, marA, marR, mdtK, msbA, rsmA, sdiA, soxR, soxS, tet(A)*	*arsS, golST, silABCDEFPRS*	*aac(6')-Iaa, bla*$_{\text{TEM-1}}$*, floR, dfrA8, tet(A), mdsA, mdsB*	*gyrB* (Gln624→Lys), *parC* (Thr57→Ser and Thr255→Ser), *acrB* (Phe28→Leu and Leu40→Pro)	-
SI 677/14	*acrA, acrB, baeR, crp, emrB, emrR, hns, kdpE, kpnF, marA, marR, mdfA, mdtK, msbA, rsmA, sdiA, soxR, soxS*	*arsR, golST*	*aac(6')-Iaa, bla*$_{\text{TEM-1}}$*, mdsA, mdsB*	*gyrB* (Gln624→Lys), *parC* (Thr57→Ser and Thr255→Ser), *acrB* (Phe28→Leu and Leu40→Pro)	IncFIB; IncFII
SI 723/14	*acrA, acrB, baeR, crp, emrB, emrR, hns, kdpE, kpnF, marA, marR, mdfA, mdtK, msbA, rsmA, sdiA, soxR, soxS*	*arsS, golST, silE*	*aac(6')-Iaa, mdsA, mdsB*	*gyrB* (Gln624→Lys), *parC* (Thr57→Ser and Thr255→Ser), *acrB* (Phe28→Leu and Leu40→Pro)	-
SI 982/14	*acrA, acrB, baeR, crp, emrB, emrR, golS, hns, kdpE, kpnF, marA, marR, mdfA, mdtK, msbA, rsmA, sdiA, soxR, soxS*	*arsR, golST*	*aac(6')-Iaa, bla*$_{\text{CMY-2}}$*, mdsA, mdsB*	*gyrB* (Gln624→Lys), *parC* (Thr57→Ser and Thr255→Ser), *acrB* (Phe28→Leu and Leu40→Pro)	IncI1-I (Alpha)
SI 1143/14	*acrA, acrB, baeR, crp, emrB, emrR, golS, hns, kdpE, kpnF, marA, marR, mdfA, mdtK, msbA, rsmA, sdiA, soxR, soxS*	*arsR, golST*	*aac(6')-Iaa, bla*$_{\text{CMY-2}}$*, mdsA, mdsB*	*gyrB* (Gln624→Lys), *parC* (Thr57→Ser and Thr255→Ser), *acrB* (Phe28→Leu and Leu40→Pro)	IncI1-I (Alpha)
SI 1284/14	*acrA, acrB, baeR, crp, emrB, emrR, golS, hns, kdpE, kpnF, marA, marR, mdtK, msbA, rsmA, sdiA, soxR, soxS, tet(A)*	*arsS, golST, silABCDEFPRS*	*aac(6')-Iaa, bla*$_{\text{TEM-1}}$*, floR, dfrA8, tet(A), mdsA, mdsB*	*gyrB* (Gln624→Lys), *parC* (Thr57→Ser and Thr255→Ser), *acrB* (Phe28→Leu and Leu40→Pro)	-
SI 1380/14	*acrA, acrB, baeR, crp, emrB, emrR, golS, hns, kdpE, kpnF, marA, marR, mdtK, msbA, rsmA, sdiA, soxR, soxS, tet(A)*	*arsR, golST*	*aac(6')-Iaa, bla*$_{\text{CMY-2}}$*, mdsA, mdsB*	*gyrB* (Gln624→Lys), *parC* (Thr57→Ser and Thr255→Ser), *acrB* (Phe28→Leu and Leu40→Pro)	IncI1-I (Alpha)
SI 1408/14	*acrA, acrB, baeR, crp, emrB, emrR, golS, hns, kdpE, kpnF, marA, marR, mdfA, mdtK, msbA, rsmA, sdiA, soxR, soxS*	*arsR, golST*	*aac(6')-Iaa, mdsA, mdsB*	*gyrB* (Gln624→Lys), *parC* (Thr57→Ser and Thr255→Ser), *acrB* (Phe28→Leu and Leu40→Pro)	-
SI 1409/14	*acrA, acrB, baeR, crp, emrB, emrR, golS, hns, kdpE, kpnF, marA, marR, mdfA, mdtK, msbA, rsmA, sdiA, soxR, soxS*	*arsR, golST*	*aac(6')-Iaa, mdsA, mdsB*	*gyrB* (Gln624→Lys), *parC* (Thr57→Ser and Thr255→Ser), *acrB* (Phe28→Leu and Leu40→Pro)	-
SI 1441/14	*acrA, acrB, baeR, crp, emrB, emrR, golS, hns, kdpE, kpnF, marA, marR, mdfA, mdtK, msbA, rsmA, sdiA, soxR, soxS*	*arsR, golST*	*aac(6')-Iaa, mdsA, mdsB*	*gyrB* (Gln624→Lys), *parC* (Thr57→Ser and Thr255→Ser), *acrB* (Phe28→Leu and Leu40→Pro)	-
SI 1711/14	*acrA, acrB, baeR, crp, emrB, emrR, golS, hns, kdpE, kpnF, marA, marR, mdfA, mdtK, msbA, rsmA, sdiA, soxR, soxS*	*arsR, golST*	*aac(6')-Iaa, bla*$_{\text{CMY-2}}$*, mdsA, mdsB*	*gyrB* (Gln624→Lys), *parC* (Thr57→Ser and Thr255→Ser), *acrB* (Phe28→Leu and Leu40→Pro)	IncI1-I (Alpha)
SI 2378/14	*acrA, acrB, baeR, crp, emrB, emrR, golS, hns, kdpE, kpnF, marA, marR, mdtK, msbA, rsmA, sdiA, soxR, soxS, tet(A)*	*arsS, golST, silABCDEFPRS*	*aac(6')-Iaa, bla*$_{\text{TEM-1}}$*, floR, dfrA8, tet(A), mdsA, mdsB*	*gyrB* (Gln624→Lys), *parC* (Thr57→Ser and Thr255→Ser), *acrB* (Phe28→Leu and Leu40→Pro)	-
SI 2430/14	*acrA, acrB, baeR, crp, emrB, emrR, golS, hns, kdpE, kpnF, marA, marR, mdtK, msbA, rsmA, sdiA,*	*arsS, golST, silABCDEFPRS*	*aac(6')-Iaa, bla*$_{\text{TEM-1}}$*, floR, dfrA8, tet(A), mdsA, mdsB*	*gyrB* (Gln624→Lys), *parC* (Thr57→Ser and Thr255→Ser), *acrB*	-

Strain no.	Efflux pump encoding genes	Heavy metal tolerance genes	Acquired resistance genes*	Chromosomal point mutations*	Plasmids*
	soxR, soxS, tet(A)			(Phe28→Leu and Leu40→Pro)	
SI 2461/14	*acrA, acrB, baeR, crp, emrB, emrR, golS, hns, kdpE, kpnF, marA, marR, mdtK, msbA, rsmA, sdiA, soxR, soxS, tet(A)*	*arsS, golST, silABCDEFPRS*	*aac(6')-Iaa*, bla_{TEM-1}, $bla_{CTX-M-8}$, *floR, dfrA8, tet(A), mdsA, mdsB*	*gyrB* (Gln624→Lys), *parC* (Thr57→Ser and Thr255→Ser), *acrB* (Phe28→Leu and Leu40→Pro)	IncI1-I (Alpha)
SI 2463/14	*acrA, acrB, baeR, crp, emrB, emrR, golS, hns, kdpE, kpnF, marA, marR, mdtK, msbA, rsmA, sdiA, soxR, soxS, tet(A)*	*arsS, golST, silABCDEFPRS*	*aac(6')-Iaa*, bla_{TEM-1}, $bla_{CTX-M-8}$, *floR, dfrA8, tet(A), mdsA, mdsB*	*gyrB* (Gln624→Lys), *parC* (Thr57→Ser and Thr255→Ser), *acrB* (Phe28→Leu and Leu40→Pro)	IncI1-I (Alpha)
SI 2548/14	*acrA, acrB, baeR, crp, emrB, emrR, golS, hns, kdpE, kpnF, marA, marR, mdfA, mdtK, msbA, rsmA, sdiA, soxR, soxS*	*arsR, golST*	*aac(6')-Iaa*, bla_{CMY-2}, *mdsA, mdsB*	*gyrB* (Gln624→Lys), *parC* (Thr57→Ser and Thr255→Ser), *acrB* (Phe28→Leu and Leu40→Pro)	IncI1-I (Alpha)
SI 3836/14	*acrA, acrB, baeR, crp, emrB, emrR, golS, hns, kdpE, kpnF, marA, marR, mdfA, mdtK, msbA, rsmA, sdiA, soxR, soxS*	*arsR, golST*	*aac(6')-Iaa, mdsA, mdsB*	*gyrB* (Gln624→Lys), *parC* (Thr57→Ser and Thr255→Ser), *acrB* (Phe28→Leu and Leu40→Pro)	-
SI 4882/14	*acrA, acrB, baeR, crp, emrB, emrR, golS, hns, kdpE, kpnF, marA, marR, mdfA, mdtK, msbA, rsmA, sdiA, soxR, soxS*	*arsR, golST*	*aac(6')-Iaa*, $bla_{CTX-M-8}$, *mdsA, mdsB*	*gyrB* (Gln624→Lys), *parC* (Thr57→Ser and Thr255→Ser), *acrB* (Phe28→Leu and Leu40→Pro)	IncI1-I (Alpha)
SI 4892/14	*acrA, acrB, baeR, crp, emrB, emrR, golS, hns, kdpE, kpnF, marA, marR, mdfA, mdtK, msbA, rsmA, sdiA, soxR, soxS*	*arsR, golST*	*aac(6')-Iaa*, $bla_{CTX-M-8}$, *mdsA, mdsB*	*gyrB* (Gln624→Lys), *parC* (Thr57→Ser and Thr255→Ser), *acrB* (Phe28→Leu and Leu40→Pro)	IncI1-I (Alpha)
SI 4895/14	*acrA, acrB, baeR, crp, emrB, emrR, golS, hns, kdpE, kpnF, marA, marR, mdfA, mdtK, msbA, rsmA, sdiA, soxR, soxS*	*arsR, golST*	*aac(6')-Iaa*, $bla_{CTX-M-8}$, *mdsA, mdsB*	*gyrB* (Gln624→Lys), *parC* (Thr57→Ser and Thr255→Ser), *acrB* (Phe28→Leu and Leu40→Pro)	IncI1-I (Alpha)
SI 4901/14	*acrA, acrB, baeR, crp, emrB, emrR, golS, hns, kdpE, kpnF, marA, marR, mdfA, mdtK, msbA, rsmA, sdiA, soxR, soxS*	*arsR, golST*	*aac(6')-Iaa*, $bla_{CTX-M-8}$, *mdsA, mdsB*	*gyrB* (Gln624→Lys), *parC* (Thr57→Ser and Thr255→Ser), *acrB* (Phe28→Leu and Leu40→Pro)	IncI1-I (Alpha)
SI 5247/14	*acrA, acrB, baeR, crp, emrB, emrR, golS, hns, kdpE, kpnF, marA, marR, mdfA, mdtK, msbA, rsmA, sdiA, soxR, soxS*	*arsR, golST*	*aac(6')-Iaa*, $bla_{CTX-M-8}$, *mdsA, mdsB*	*gyrB* (Gln624→Lys), *parC* (Thr57→Ser and Thr255→Ser), *acrB* (Phe28→Leu and Leu40→Pro)	IncI1-I (Alpha)
SI 342/15	*acrA, acrB, baeR, crp, emrB, emrR, golS, hns, kdpE, kpnF, marA, marR, mdtK, msbA, rsmA, sdiA, soxR, soxS, tet(A)*	*arsS, golST, silABCDEFPRS*	*aac(6')-Iaa*, bla_{TEM-1}, *floR, dfrA8, tet(A), mdsA, mdsB*	*gyrB* (Gln624→Lys), *parC* (Thr57→Ser and Thr255→Ser), *acrB* (Phe28→Leu and Leu40→Pro)	-
SI 444/15	*acrA, acrB, baeR, crp, emrB, emrR, golS, hns, kdpE, kpnF, marA, marR, mdtK, msbA, rsmA, sdiA, soxR, soxS, tet(A)*	*arsS, golST, silABCDEFPRS*	*aac(6')-Iaa*, bla_{TEM-1}, *floR, dfrA8, tet(A), mdsA, mdsB*	*gyrB* (Gln624→Lys), *parC* (Thr57→Ser and Thr255→Ser), *acrB* (Phe28→Leu and Leu40→Pro)	-
SI 447/15	*acrA, acrB, baeR, crp, emrB, emrR, golS, hns, kdpE, kpnF, marA, marR, mdtK, msbA, rsmA, sdiA, soxR, soxS, tet(A)*	*arsS, golST, silABCDEFPRS*	*aac(6')-Iaa*, bla_{TEM-1}, *floR, dfrA8, tet(A), mdsA, mdsB*	*gyrB* (Gln624→Lys), *parC* (Thr57→Ser and Thr255→Ser), *acrB* (Phe28→Leu and Leu40→Pro)	-

Strain no.	Efflux pump encoding genes	Heavy metal tolerance genes	Acquired resistance genes*	Chromosomal point mutations*	Plasmids*
SI 1809/15	*acrA, acrB, baeR, crp, emrB, emrR, golS, hns, kdpE, kpnF, marA, marR, mdtK, msbA, rsmA, sdiA, soxR, soxS, tet(A)*	*arsS, golST, silABCDEFPRS*	*aac(6')-Iaa, bla*$_{TEM-1}$*, floR, dfrA8, tet(A), mdsA, mdsB*	*gyrB* (Gln624→Lys), *parC* (Thr57→Ser and Thr255→Ser), *acrB* (Phe28→Leu and Leu40→Pro)	-
SI 1816/15	*acrA, acrB, baeR, crp, emrB, emrR, golS, hns, kdpE, kpnF, marA, marR, mdtK, msbA, rsmA, sdiA, soxR, soxS, tet(A)*	*arsS, golST, silABCDEFPRS*	*aac(6')-Iaa, bla*$_{TEM-1}$*, floR, dfrA8, tet(A), mdsA, mdsB*	*gyrB* (Gln624→Lys), *parC* (Thr57→Ser and Thr255→Ser), *acrB* (Phe28→Leu and Leu40→Pro)	IncQ1
SI 2280/15	*acrA, acrB, baeR, crp, emrB, emrR, golS, hns, kdpE, kpnF, marA, marR, mdtK, msbA, rsmA, sdiA, soxR, soxS, tet(A)*	*arsS, golST, silABCDEFPRS*	*aac(6')-Iaa, bla*$_{TEM-1}$*, floR, dfrA8, tet(A), mdsA, mdsB*	*gyrB* (Gln624→Lys), *parC* (Thr57→Ser and Thr255→Ser), *acrB* (Phe28→Leu and Leu40→Pro)	-
SI 2302/15	*acrA, acrB, baeR, crp, emrB, emrR, golS, hns, kdpE, kpnF, marA, marR, mdfA, mdtK, msbA, rsmA, sdiA, soxR, soxS*	*arsR, golST*	*aac(6')-Iaa, mdsA, mdsB*	*gyrB* (Gln624→Lys), *parC* (Thr57→Ser and Thr255→Ser), *acrB* (Phe28→Leu and Leu40→Pro)	-
SI 2370/15	*acrA, acrB, baeR, crp, emrB, emrR, golS, hns, kdpE, kpnF, marA, marR, mdfA, mdtK, msbA, rsmA, sdiA, soxR, soxS*	*arsR, golST*	*aac(6')-Iaa, mdsA, mdsB*	*gyrB* (Gln624→Lys), *parC* (Thr57→Ser and Thr255→Ser), *acrB* (Phe28→Leu and Leu40→Pro)	-
SI 2869/15	*acrA, acrB, baeR, crp, emrB, emrR, golS, hns, kdpE, kpnF, marA, marR, mdfA, mdtK, msbA, rsmA, sdiA, soxR, soxS*	*arsR, golST*	*aac(6')-Iaa, mdsA, mdsB*	*gyrB* (Gln624→Lys), *parC* (Thr57→Ser and Thr255→Ser), *acrB* (Phe28→Leu and Leu40→Pro)	-
SI 3056/15	*acrA, acrB, baeR, crp, emrB, emrR, golS, hns, kdpE, kpnF, marA, marR, mdfA, mdtK, msbA, rsmA, sdiA, soxR, soxS*	*arsR, golST*	*aac(6')-Iaa, bla*$_{CTX-M-8}$*, mdsA, mdsB*	*gyrB* (Gln624→Lys), *parC* (Thr57→Ser and Thr255→Ser), *acrB* (Phe28→Leu and Leu40→Pro)	IncI1-I (Alpha)
SI 4764/15	*acrA, acrB, baeR, crp, emrB, emrR, golS, hns, kdpE, kpnF, marA, marR, mdtK, msbA, rsmA, sdiA, soxR, soxS, tet(A)*	*arsS, golST, silABCDEFPRS*	*aac(6')-Iaa, bla*$_{TEM-1}$*, floR, dfrA8, tet(A), mdsA, mdsB*	*gyrB* (Gln624→Lys), *parC* (Thr57→Ser and Thr255→Ser), *acrB* (Phe28→Leu and Leu40→Pro)	-
SI 5391/15	*acrA, acrB, baeR, crp, emrB, emrR, golS, hns, kdpE, kpnF, marA, marR, mdfA, mdtK, msbA, rsmA, sdiA, soxR, soxS*	*arsR, golST*	*aac(6')-Iaa, bla*$_{CMY-2}$*, mdsA, mdsB*	*gyrB* (Gln624→Lys), *parC* (Thr57→Ser and Thr255→Ser), *acrB* (Phe28→Leu and Leu40→Pro)	IncI1-I (Alpha)
SI 5837/15	*acrA, acrB, baeR, crp, emrB, emrR, golS, hns, kdpE, kpnF, marA, marR, mdfA, mdtK, msbA, rsmA, sdiA, soxR, soxS*	*arsR, golST*	*aac(6')-Iaa, mdsA, mdsB*	*gyrB* (Gln624→Lys), *parC* (Thr57→Ser and Thr255→Ser), *acrB* (Phe28→Leu and Leu40→Pro)	-
SI 5853/15	*acrA, acrB, baeR, crp, emrB, emrR, golS, hns, kdpE, kpnF, marA, marR, mdfA, mdtK, msbA, rsmA, sdiA, soxR, soxS*	*arsR, golST*	*aac(6')-Iaa, mdsA, mdsB*	*gyrB* (Gln624→Lys), *parC* (Thr57→Ser and Thr255→Ser), *acrB* (Phe28→Leu and Leu40→Pro)	-
SI 5859/15	*acrA, acrB, baeR, crp, emrB, emrR, golS, hns, kdpE, kpnF, marA, marR, mdfA, mdtK, msbA, rsmA, sdiA, soxR, soxS*	*arsR, golST*	*aac(6')-Iaa, mdsA, mdsB*	*gyrB* (Gln624→Lys), *parC* (Thr57→Ser and Thr255→Ser), *acrB* (Phe28→Leu and Leu40→Pro)	-
SI 5911/15	*acrA, acrB, baeR, crp, emrB, emrR, golS, hns, kdpE, kpnF, marA, marR, mdtK, msbA, rsmA, sdiA,*	*arsS, golST, silABCDEFPRS*	*aac(6')-Iaa, bla*$_{TEM-1}$*, floR, dfrA8, tet(A), mdsA, mdsB*	*gyrB* (Gln624→Lys), *parC* (Thr57→Ser and Thr255→Ser),	-

Strain no.	Efflux pump encoding genes	Heavy metal tolerance genes	Acquired resistance genes*	Chromosomal point mutations*	Plasmids*
	soxR, soxS, tet(A)			*acrB* (Phe28→Leu and Leu40→Pro)	
SI 5912/15	*acrA, acrB, baeR, crp, emrB, emrR, golS, hns, kdpE, kpnF, marA, marR, mdtK, msbA, rsmA, sdiA, soxR, soxS, tet(A)*	*arsS, golST, silABCDEFPRS*	*aac(6')-Iaa, bla*$_{TEM-1}$*, floR, dfrA8, tet(A), mdsA, mdsB*	*gyrB* (Gln624→Lys), *parC* (Thr57→Ser and Thr255→Ser), *acrB* (Phe28→Leu and Leu40→Pro)	-
SI 5915/15	*acrA, acrB, baeR, crp, emrB, emrR, golS, hns, kdpE, kpnF, marA, marR, mdtK, msbA, rsmA, sdiA, soxR, soxS, tet(A)*	*arsS, golST, silABCDEFPRS*	*aac(6')-Iaa, bla*$_{TEM-1}$*, floR, dfrA8, tet(A), mdsA, mdsB*	*gyrB* (Gln624→Lys), *parC* (Thr57→Ser and Thr255→Ser), *acrB* (Phe28→Leu and Leu40→Pro)	-
SI 5923/15	*acrA, acrB, baeR, crp, emrB, emrR, golS, hns, kdpE, kpnF, marA, marR, mdtK, msbA, rsmA, sdiA, soxR, soxS, tet(A)*	*arsS, golST, silABCDEFPRS*	*aac(6')-Iaa, bla*$_{TEM-1}$*, floR, dfrA8, tet(A), mdsA, mdsB*	*gyrB* (Gln624→Lys), *parC* (Thr57→Ser and Thr255→Ser), *acrB* (Phe28→Leu and Leu40→Pro)	-
SI 220/16	*acrA, acrB, baeR, crp, emrB, emrR, golS, hns, kdpE, kpnF, marA, marR, mdtK, msbA, rsmA, sdiA, soxR, soxS, tet(A)*	*arsS, golST, silABCDEFPRS*	*aac(6')-Iaa, bla*$_{TEM-1}$*, floR, dfrA8, tet(A), mdsA, mdsB*	*gyrB* (Gln624→Lys), *parC* (Thr57→Ser and Thr255→Ser), *acrB* (Phe28→Leu and Leu40→Pro)	-
SI 3687/16	*acrA, acrB, baeR, crp, emrB, emrR, golS, hns, kdpE, kpnF, marA, marR, mdtK, msbA, rsmA, sdiA, soxR, soxS, tet(A)*	*arsS, golST, silABCDEFPRS*	*aac(6')-Iaa, bla*$_{TEM-1}$*, floR, dfrA8, tet(A), mdsA, mdsB*	*gyrB* (Gln624→Lys), *parC* (Thr57→Ser and Thr255→Ser), *acrB* (Phe28→Leu and Leu40→Pro)	IncI1-I (Alpha); IncN; IncR
SI 4447/16	*acrA, acrB, baeR, crp, emrB, emrR, golS, hns, kdpE, kpnF, marA, marR, mdtK, msbA, rsmA, sdiA, soxR, soxS, tet(A)*	*arsS, golST, silABCDEFPRS*	*aac(6')-Iaa, bla*$_{TEM-1}$*, floR, dfrA8, tet(A), mdsA, mdsB*	*gyrB* (Gln624→Lys), *parC* (Thr57→Ser and Thr255→Ser), *acrB* (Phe28→Leu and Leu40→Pro)	-
SI 5946/16	*acrA, acrB, baeR, crp, emrB, emrR, golS, hns, kdpE, kpnF, marA, marR, mdtK, msbA, rsmA, sdiA, soxR, soxS, tet(A)*	*arsS, golST, silABCDEFPRS*	*aac(6')-Iaa, bla*$_{TEM-1}$*, floR, dfrA8, tet(A), mdsA, mdsB*	*gyrB* (Gln624→Lys), *parC* (Thr57→Ser and Thr255→Ser), *acrB* (Phe28→Leu and Leu40→Pro)	-
SI 6987/16	*acrA, acrB, baeR, crp, emrB, emrR, golS, hns, kdpE, kpnF, marA, marR, mdfA, mdtK, msbA, rsmA, sdiA, soxR, soxS*	*arsR, golST*	*aph(3'')-Ib, aph(6)-Id, aac(6')-Iaa, bla*$_{TEM-1}$*, dfrA8, sul2, mdsA, mdsB*	*gyrB* (Gln624→Lys), *parC* (Thr57→Ser and Thr255→Ser), *acrB* (Phe28→Leu and Leu40→Pro)	IncFII(29)
SI 7876/16	*acrA, acrB, baeR, crp, emrB, emrR, golS, hns, kdpE, kpnF, marA, marR, mdtK, msbA, rsmA, sdiA, soxR, soxS, tet(A)*	*arsS, golST, silABCDEFPRS*	*aac(6')-Iaa, bla*$_{TEM-1}$*, bla*$_{CTX-M-8}$*, floR, dfrA8, tet(A), mdsA, mdsB*	*gyrB* (Gln624→Lys), *parC* (Thr57→Ser and Thr255→Ser), *acrB* (Phe28→Leu and Leu40→Pro)	IncI1-I (Alpha)
SI 11/17	*acrA, acrB, baeR, crp, emrB, emrR, golS, hns, kdpE, kpnF, marA, marR, mdfA, mdtK, msbA, rsmA, sdiA, soxR, soxS*	*arsR, golST*	*aac(6')-Iaa, mdsA, mdsB*	*gyrB* (Gln624→Lys), *parC* (Thr57→Ser and Thr255→Ser), *acrB* (Phe28→Leu and Leu40→Pro)	-
SI 23/17	*acrA, acrB, baeR, crp, emrB, emrR, golS, hns, kdpE, kpnF, marA, marR, mdfA, mdtK, msbA, rsmA, sdiA, soxR, soxS*	*arsR, golST*	*aac(6')-Iaa, mdsA, mdsB*	*gyrB* (Gln624→Lys), *parC* (Thr57→Ser and Thr255→Ser), *acrB* (Phe28→Leu and Leu40→Pro)	-
SI 238/17	*acrA, acrB, baeR, crp, emrB, emrR, golS, hns, kdpE, kpnF, marA, marR, mdfA, mdtK, msbA, rsmA, sdiA, soxR, soxS*	*arsR, golST*	*aac(6')-Iaa, mdsA, mdsB*	*gyrB* (Gln624→Lys), *parC* (Thr57→Ser and Thr255→Ser), *acrB* (Phe28→Leu and Leu40→Pro)	-

Strain no.	Efflux pump encoding genes	Heavy metal tolerance genes	Acquired resistance genes*	Chromosomal point mutations*	Plasmids*
SI 872/17	*acrA, acrB, baeR, crp, emrB, emrR, golS, hns, kdpE, kpnF, marA, marR, mdfA, mdtK, msbA, rsmA, sdiA, soxR, soxS*	*arsR, golST*	*aac(6')-Iaa, mdsA, mdsB*	*gyrB* (Gln624→Lys), *parC* (Thr57→Ser and Thr255→Ser), *acrB* (Phe28→Leu and Leu40→Pro)	-
SI 1171/17	*acrA, acrB, baeR, crp, emrB, emrR, golS, hns, kdpE, kpnF, marA, marR, mdfA, mdtK, msbA, rsmA, sdiA, soxR, soxS*	*arsR, golST*	*aac(6')-Iaa, mdsA, mdsB*	*gyrB* (Gln624→Lys), *parC* (Thr57→Ser and Thr255→Ser), *acrB* (Phe28→Leu and Leu40→Pro)	-
SI 1256/17	*acrA, acrB, baeR, crp, emrB, emrR, golS, hns, kdpE, kpnF, marA, marR, mdfA, mdtK, msbA, rsmA, sdiA, soxR, soxS*	*arsR, golST*	*aac(6')-Iaa, mdsA, mdsB*	*gyrB* (Gln624→Lys), *parC* (Thr57→Ser and Thr255→Ser), *acrB* (Phe28→Leu and Leu40→Pro)	-
SI 2580/17	*acrA, acrB, baeR, crp, emrB, emrR, golS, hns, kdpE, kpnF, marA, marR, mdfA, mdtK, msbA, rsmA, sdiA, soxR, soxS*	*arsR, golST*	*aac(6')-Iaa, mdsA, mdsB*	*gyrB* (Gln624→Lys), *parC* (Thr57→Ser, Thr255→Ser and Val702-Ala), *acrB* (Phe28→Leu and Leu40→Pro)	-
SI 2953/17	*acrA, acrB, baeR, crp, emrB, emrR, golS, hns, kdpE, kpnF, marA, marR, mdfA, mdtK, msbA, rsmA, sdiA, soxR, soxS*	*arsR, golST*	*aac(6')-Iaa, mdsA, mdsB*	*gyrB* (Gln624→Lys), *parC* (Thr57→Ser and Thr255→Ser), *acrB* (Phe28→Leu and Leu40→Pro)	-
SI 2954/17	*acrA, acrB, baeR, crp, emrB, emrR, golS, hns, kdpE, kpnF, marA, marR, mdfA, mdtK, msbA, rsmA, sdiA, soxR, soxS*	*arsR, golST*	*aac(6')-Iaa, mdsA, mdsB*	*gyrB* (Gln624→Lys), *parC* (Thr57→Ser and Thr255→Ser), *acrB* (Phe28→Leu and Leu40→Pro)	-
SI 3380/17	*acrA, acrB, baeR, crp, emrB, emrR, golS, hns, kdpE, kpnF, marA, marR, mdfA, mdtK, msbA, rsmA, sdiA, soxR, soxS*	*arsR, golST*	*aac(6')-Iaa, mdsA, mdsB*	*gyrB* (Gln624→Lys), *parC* (Thr57→Ser and Thr255→Ser), *acrB* (Phe28→Leu and Leu40→Pro)	-
SI 3877/17	*acrA, acrB, baeR, crp, emrB, emrR, golS, hns, kdpE, kpnF, marA, marR, mdfA, mdtK, msbA, rsmA, sdiA, soxR, soxS*	*arsR, golST*	*aac(6')-Iaa, mdsA, mdsB*	*gyrB* (Gln624→Lys), *parC* (Thr57→Ser and Thr255→Ser), *acrB* (Phe28→Leu and Leu40→Pro)	-
SI 3906/17	*acrA, acrB, baeR, crp, emrB, emrR, golS, hns, kdpE, kpnF, marA, marR, mdtK, msbA, rsmA, sdiA, soxR, soxS, tet(A)*	*arsS, golST, silABCDEFPRS*	*aac(6')-Iaa,* bla_{TEM-1}, *floR, dfrA8, tet(A), mdsA, mdsB*	*gyrB* (Gln624→Lys), *parC* (Thr57→Ser and Thr255→Ser), *acrB* (Phe28→Leu and Leu40→Pro)	-
SI 4065/17	*acrA, acrB, baeR, crp, emrB, emrR, golS, hns, kdpE, kpnF, marA, marR, mdtK, msbA, rsmA, sdiA, soxR, soxS, tet(A)*	*arsS, golST, silABCDEFPRS*	*aac(6')-Iaa,* bla_{TEM-1}, *floR, dfrA8, tet(A), mdsA, mdsB*	*gyrB* (Gln624→Lys), *parC* (Thr57→Ser and Thr255→Ser), *acrB* (Phe28→Leu and Leu40→Pro)	-
SI 4067/17	*acrA, acrB, baeR, crp, emrB, emrR, golS, hns, kdpE, kpnF, marA, marR, mdtK, msbA, rsmA, sdiA, soxR, soxS, tet(A)*	*arsS, golST, silABCDEFPRS*	*aac(6')-Iaa,* bla_{TEM-1}, *floR, dfrA8, tet(A), mdsA, mdsB*	*gyrB* (Gln624→Lys), *parC* (Thr57→Ser and Thr255→Ser), *acrB* (Phe28→Leu and Leu40→Pro)	-
SI 4069/17	*acrA, acrB, baeR, crp, emrB, emrR, golS, hns, kdpE, kpnF, marA, marR, mdfA, mdtK, msbA, rsmA, sdiA, soxR, soxS*	*arsR, golST*	*aac(6')-Iaa, mdsA, mdsB*	*gyrB* (Gln624→Lys), *parC* (Thr57→Ser and Thr255→Ser), *acrB* (Phe28→Leu and Leu40→Pro)	-

Strain no.	Efflux pump encoding genes	Heavy metal tolerance genes	Acquired resistance genes*	Chromosomal point mutations*	Plasmids*
SI 52/18	*acrA, acrB, baeR, crp, emrB, emrR, golS, hns, kdpE, kpnF, marA, marR, mdfA, mdtK, msbA, rsmA, sdiA, soxR, soxS*	*arsR, golST*	*aac(6')-Iaa, bla*$_{CTX-M-8}$, *mdsA, mdsB*	*gyrB* (Gln624→Lys), *parC* (Thr57→Ser and Thr255→Ser), *acrB* (Phe28→Leu and Leu40→Pro)	IncI1-I (Alpha)
SI 331/18	*acrA, acrB, baeR, crp, emrB, emrR, golS, hns, kdpE, kpnF, marA, marR, mdfA, mdtK, msbA, rsmA, sdiA, soxR, soxS*	*arsR, golST*	*aac(6')-Iaa, mdsA, mdsB*	*gyrB* (Gln624→Lys), *parC* (Thr57→Ser and Thr255→Ser), *acrB* (Phe28→Leu and Leu40→Pro)	-
SI 623/18	*acrA, acrB, baeR, crp, emrB, emrR, golS, hns, kdpE, kpnF, marA, marR, mdfA, mdtK, msbA, rsmA, sdiA, soxR, soxS*	*arsR, golST*	*aac(6')-Iaa, mdsA, mdsB*	*gyrB* (Gln624→Lys), *parC* (Thr57→Ser and Thr255→Ser), *acrB* (Phe28→Leu and Leu40→Pro)	-
SI 661/18	*acrA, acrB, baeR, crp, emrB, emrR, golS, hns, kdpE, kpnF, marA, marR, mdfA, mdtK, msbA, rsmA, sdiA, soxR, soxS*	*arsR, golST*	*aac(6')-Iaa, mdsA, mdsB*	*gyrB* (Gln624→Lys), *parC* (Thr57→Ser and Thr255→Ser), *acrB* (Phe28→Leu and Leu40→Pro)	-
SI 942/18	*acrA, acrB, baeR, crp, emrB, emrR, golS, hns, kdpE, kpnF, marA, marR, mdtK, msbA, rsmA, sdiA, soxR, soxS, tet(A)*	*arsS, golST, silABCDEFPRS*	*aac(6')-Iaa, bla*$_{TEM-1}$, *floR, dfrA8, tet(A), mdsA, mdsB*	*gyrB* (Gln624→Lys), *parC* (Thr57→Ser and Thr255→Ser), *acrB* (Phe28→Leu and Leu40→Pro)	-
SI 1634/18	*acrA, acrB, baeR, crp, emrB, emrR, golS, hns, kdpE, kpnF, marA, marR, mdfA, mdtK, msbA, rsmA, sdiA, soxR, soxS*	*arsR, golST*	*aac(6')-Iaa, mdsA, mdsB*	*gyrB* (Gln624→Lys), *parC* (Thr57→Ser and Thr255→Ser), *acrB* (Phe28→Leu and Leu40→Pro)	-
SI 2676/18	*acrA, acrB, baeR, crp, emrB, emrR, golS, hns, kdpE, kpnF, marA, marR, mdfA, mdtK, msbA, rsmA, sdiA, soxR, soxS*	*arsR, golST*	*aac(6')-Iaa, bla*$_{CMY-2}$, *mdsA, mdsB*	*gyrB* (Gln624→Lys), *parC* (Thr57→Ser and Thr255→Ser), *acrB* (Phe28→Leu and Leu40→Pro)	IncI1-I (Alpha); IncX1

* This information regarding acquired resistance genes, chromosomal point mutations and plasmids have been detailedly published in Vilela et al. (2022) J. Applied Micro 132, 3327–3342. https://doi.org/10.1111/jam.15430
