# Supplementary material for: Prevalence of efflux pump and heavy metal tolerance encoding genes among Salmonella enterica serovar Infantis strains from diverse sources in Brazil
Source: PLoS One. 2022 Nov 22;17(11):e0277979. doi: 10.1371/journal.pone.0277979 (PMC9681071; doi:10.1371/journal.pone.0277979)
Supplement: S3 Table — (PDF) [file pone.0277979.s003.pdf]

Table S3 - List of the drug classes and resistance mechanisms as referred by the Comprehensive Antibiotic Resistance Database (CARD) of the efflux pump encoding genes detected among the 80 sequenced *Salmonella* Infantis strains studied isolated from food (n=27), farm and industry environments (n=24), humans (n=19), animals (n=7) and animal feed (n=3) in Brazil between 2013 and 2018.

Gene	Drug class	Resistance mechanism
acrA	Quinolones, cephalosporins, glycylcyclines, penams, tetracyclines, rifamycins, phenicols, triclosan	Antibiotic efflux
acrB	Quinolones, cephalosporins, glycylcyclines, penams, tetracyclines, rifamycins, phenicols, triclosan	Antibiotic efflux
baeR	Aminoglycosides, aminocoumarins	Antibiotic efflux
crp	Macrolides, quinolones, penams	Antibiotic efflux
emrB	Quinolones	Antibiotic efflux
emrR	Quinolones	Antibiotic efflux
golS	Monobactams, carbapenems, cephalosporins, cephamycins, penams, phenicols, penems	Antibiotic efflux
hns	Macrolides, quinolones, cephalosporins, cephamycins, penams, tetracyclines	Antibiotic efflux
kdpE	Aminoglycosides	Antibiotic efflux
kpnF	Macrolides, aminoglycosides, cephalosporins, tetracyclines, antibiotic peptides, rifamycins	Antibiotic efflux
marA	Quinolones, monobactams, carbapenems, cephalosporins, glycylcyclines, cephamycins, penams, tetracyclines, rifamycins, phenicols, triclosan, penems	Antibiotic efflux and reduced permeability
marR	Quinolones, cephalosporins, glycylcyclines, penams, tetracyclines, rifamycins, phenicols, triclosan	Antibiotic efflux and target alteration
mdfA	Tetracyclines, benzalkonium chloride, rhodamine	Antibiotic efflux
mdtK	Quinolones	Antibiotic efflux
msbA	Nitroimidazoles	Antibiotic efflux
rsmA	Quinolones, diaminopyrimidine compounds, phenicols	Antibiotic efflux
sdiA	Quinolones, cephalosporins, glycylcyclines, penams, tetracyclines, rifamycins, phenicols, triclosan	Antibiotic efflux
soxR	Quinolones, cephalosporins, glycylcyclines, penams, tetracyclines, rifamycins, phenicols, triclosan	Antibiotic efflux and target alteration
soxS	Quinolones, monobactams, carbapenems, cephalosporins, glycylcyclines, cephamycins, penams, tetracyclines, rifamycins, phenicols, triclosan, penems	Antibiotic efflux, target alteration and reduced permeability
tet(A)	Tetracyclines	Antibiotic efflux
