# Supplementary material for: Prevalence of efflux pump and heavy metal tolerance encoding genes among Salmonella enterica serovar Infantis strains from diverse sources in Brazil
Source: PLoS One. 2022 Nov 22;17(11):e0277979. doi: 10.1371/journal.pone.0277979 (PMC9681071; doi:10.1371/journal.pone.0277979)
Supplement: S1 Fig — (PDF) [file pone.0277979.s004.pdf]

Figure S1 – Statistical analysis based on the Chi-Square test to verify the association among the 46 *S.* Infantis strains co-harboring *golS*, *golT*, *arsR* and the profile 1 of efflux pump encoding genes (A1 and B1) and 29 strains harboring *golS*, *golT*, *arsR*, the *sil* operon and the profile 2 of efflux pump encoding genes (A2 and B2). Figures A1 and A2 represent the graphic and B1 and B2 the tabular results, obtained with the software GraphPad Prism 5.

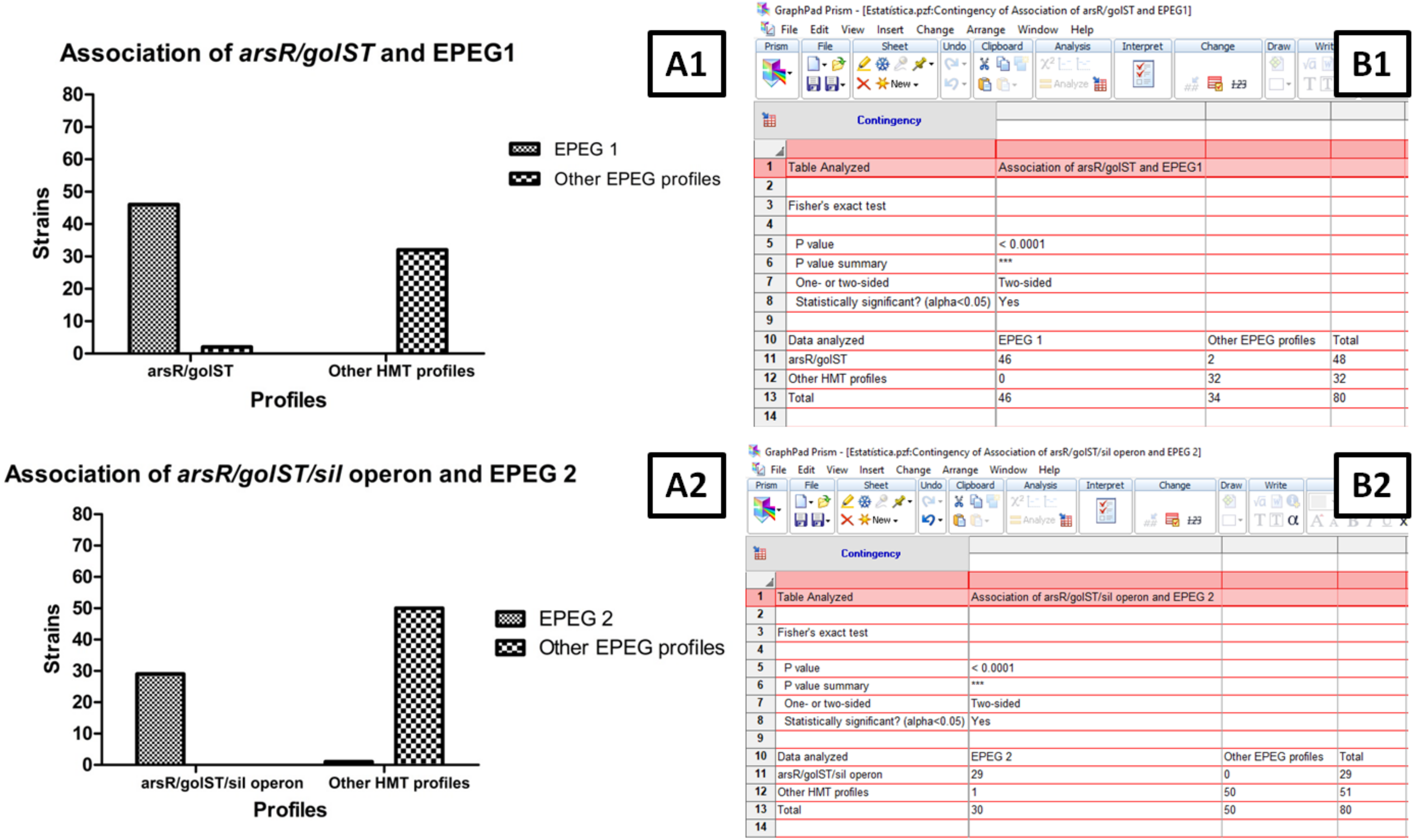

1	Table Analyzed	Association of arsR/golST and EPEG1		
2				
3	Fisher's exact test			
4				
5	P value	< 0.0001		
6	P value summary	***		
7	One- or two-sided	Two-sided		
8	Statistically significant? (alpha<0.05)	Yes		
9				
10	Data analyzed	EPEG 1	Other EPEG profiles	Total
11	arsR/golST	46	2	48
12	Other HMT profiles	0	32	32
13	Total	46	34	80
14				

1	Table Analyzed	Association of arsR/golST/sil operon and EPEG 2		
2				
3	Fisher's exact test			
4				
5	P value	< 0.0001		
6	P value summary	***		
7	One- or two-sided	Two-sided		
8	Statistically significant? (alpha<0.05)	Yes		
9				
10	Data analyzed	EPEG 2	Other EPEG profiles	Total
11	arsR/golST/sil operon	29	0	29
12	Other HMT profiles	1	50	51
13	Total	30	50	80
14				

EPEG, efflux pump encoding genes; HMT, heavy metal tolerance encoding genes

EPEG 1: *acrA, acrB, baeR, crp, emrB, emrR, golS, hns, kdpE, kpnF, marA, marR, mdfA, mdtK, msbA, rsmA, sdiA, soxR, soxS*

EPEG 2: *acrA, acrB, baeR, crp, emrB, emrR, golS, hns, kdpE, kpnF, marA, marR, mdtK, msbA, rsmA, sdiA, soxR, soxS, tet(A)*
